# Impaired firing properties of dentate granule neurons in an Alzheimer's disease animal model are rescued by PPARγ agonism

**DOI:** 10.1152/jn.00419.2014

**Published:** 2014-12-24

**Authors:** Miroslav N. Nenov, Filippo Tempia, Larry Denner, Kelly T. Dineley, Fernanda Laezza

**Affiliations:** ^1^Department of Pharmacology and Toxicology, The University of Texas Medical Branch, Galveston, Texas;; ^2^Department of Neurology, The University of Texas Medical Branch, Galveston, Texas;; ^3^Department of Internal Medicine, The University of Texas Medical Branch, Galveston, Texas;; ^4^Center for Addiction Research, The University of Texas Medical Branch, Galveston, Texas;; ^5^Mitchell Center for Neurodegenerative Diseases, The University of Texas Medical Branch, Galveston, Texas; and; ^6^Center for Biomedical Engineering, The University of Texas Medical Branch, Galveston, Texas

**Keywords:** Alzheimer's disease, dentate gyrus, excitability, patch clamp, peroxisome proliferator-activated receptor-γ

## Abstract

Early cognitive impairment in Alzheimer's disease (AD) correlates with medial temporal lobe dysfunction, including two areas essential for memory formation: the entorhinal cortex and dentate gyrus (DG). In the Tg2576 animal model for AD amyloidosis, activation of the peroxisome proliferator-activated receptor-gamma (PPARγ) with rosiglitazone (RSG) ameliorates hippocampus-dependent cognitive impairment and restores aberrant synaptic activity at the entorhinal cortex to DG granule neuron inputs. It is unknown, however, whether intrinsic firing properties of DG granule neurons in these animals are affected by amyloid-β pathology and if they are sensitive to RSG treatment. Here, we report that granule neurons from 9-mo-old wild-type and Tg2576 animals can be segregated into two cell types with distinct firing properties and input resistance that correlate with less mature type I and more mature type II neurons. The DG type I cell population was greater than type II in wild-type littermates. In the Tg2576 animals, the type I and type II cell populations were nearly equal but could be restored to wild-type levels through cognitive enhancement with RSG. Furthermore, Tg2576 cell firing frequency and spike after depolarization were decreased in type I and increased in type II cells, both of which could also be restored to wild-type levels upon RSG treatment. That these parameters were restored by PPARγ activation emphasizes the therapeutic value of RSG against early AD cognitive impairment.

the dentate gyrus (DG) is critical for memory formation and serves as the first integrative weigh station in the hippocampal trisynaptic circuit. Through complex signal processing, the DG converts extrahippocampal information into spatiotemporal patterns of activity (Krueppel et al. 2011), which are projected along the hippocampal circuit and stored as memory engrams during memory consolidation (Rolls and Kesner 2006). It is thought that continuous generation, differentiation, and maturation of new granule neurons, which for several months remain highly excitable and display enhanced synaptic plasticity, are required for normal cognitive function (Bruel-Jungerman et al. 2009; Ge et al. 2007; Schmidt-Hieber et al. 2004). Dysfunction of the DG circuit is considered one of the earliest functional alterations associated with Alzheimer's disease (AD) cognitive impairment (Palmer and Good 2011). Ours and others' work using mouse models for AD amyloidosis discovered that early AD cognitive impairment is accompanied by changes in the entorhinal cortex to DG synaptic transmission (Nenov et al. 2014) and epileptic-like hyperexcitability of the DG circuit (Hazra et al. 2013; Palop et al. 2007; Ziburkus et al. 2013), possibly through dysregulation of the presynaptic vesicle-release machinery (Nenov et al. 2014), voltage-dependent ion channels (Verret et al. 2012), or the ERK-MAPK signaling pathway (Denner et al. 2012; Dineley et al. 2001, 2002). Emerging views suggest that these early, aberrant changes within the DG circuit might result from depletion or inappropriate maturation of adult-born granule neurons (Shruster et al. 2010), culminating in a more global loss of synaptic function that underlies the severe memory loss seen at a later disease stage.

Intrinsic firing and membrane properties reflect the level of integration of adult-born granule neurons of the DG circuit that greatly influences memory engram formation (Rolls and Kesner 2006). Alterations of firing properties in granule neurons might therefore be one of the first functional phenotypes detected in early AD preceding AD synaptic deficits and memory dysfunction (Cuevas et al. 2011; Palmer and Good 2011; Rowan et al. 2005; Trommer et al. 2005). Thus the restoration of intrinsic properties of DG granule neurons might be a strategy for early AD prevention and treatment.

A promising therapeutic strategy for early AD cognitive impairment is treatment with the thiazolidinediones pioglitazone and rosiglitazone (RSG), potent and selective agonists of peroxisome proliferator-activated receptor-gamma (PPARγ) (Dickerson and Eichenbaum 2010; Harrington et al. 2007; Risner et al. 2006; Watson et al. 2005). PPARγ is a type II nuclear receptor with well-characterized functions in restoring insulin sensitivity in type 2 diabetes. Since insulin resistance and diabetes are now known risk factors for AD, RSG has been tested for its memory-enhancing ability in animal models and AD patients with some success (Denner et al. 2012; Dickerson and Eichenbaum 2010; Harrington et al. 2007; Jahrling et al. 2014; Risner et al. 2006; Rodriguez-Rivera et al. 2011; Watson et al. 2005). However, the neural circuitry mechanisms underlying the central PPARγ agonism as a cognitive-enhancing approach remain poorly understood, limiting full exploitation of insulin sensitizers for AD applications.

In previous studies, we demonstrated that presynaptic inputs from the medial perforant path (mPP) to DG neurons in the Tg2576 animal model of AD are upregulated and exhibit impaired, short-term plasticity and that 1-mo dietary treatment with RSG normalizes these alterations (Nenov et al. 2014). In this study, we postulated that in addition to synaptic rescue, activation of PPARγ would restore dysfunctions of the DG granule cell firing properties in the Tg2576 AD mouse model that might complement or precede synaptic deficits.

With the application of whole-cell patch-clamp electrophysiological recordings in DG granule neurons from 9-mo-old wild-type and Tg2576 mice, with and without RSG, we show that amyloid-β (Aβ) pathology skews the DG neuron population from a predominantly less mature cell phenotype to a more mature cell phenotype. Along with that, cell type-specific aberrant firing properties and spike after-depolarization potentials (ADPs) were found to be induced upon Aβ pathology. The relative proportions of DG granule neuron types and the changes in intrinsic firing properties and ADP values were rescued by RSG treatment. Our results fill a significant knowledge gap in the cellular mechanisms underlying early AD manifestation and provide an important functional link between cognitive enhancement through PPARγ agonism and DG granule neuron function, opening new avenues for AD therapy development.

## METHODS

### 

#### Animals.

Animals were bred in The University of Texas Medical Branch (UTMB) Animal Care Facility by mating heterozygous Tg2576 male with (C57BL6/J × SJL/J)F1 female mice (The Jackson Laboratory, Bar Harbor, ME). UTMB operates in compliance with the U.S. Department of Agriculture Animal Welfare Act, the *Guide for the Care and Use of Laboratory Animals*, and Institutional Animal Care and Use Committee-approved protocols. Mice were housed (*n* ≤ 5/cage) with food and water ad libitum. All animal manipulations were conducted during the lights-on phase (0700–1900). Male and female 8-mo-old Tg2576 and wild-type littermates were fed with a control or a 30-mg/kg RSG diet (Bio-Serv, Flemington, NJ) for 30 days, as described previously (Nenov et al. 2014; Rodriguez-Rivera et al. 2011).

#### Slice preparation.

Acute hippocampal slices were prepared from 9-mo-old wild-type littermate control and Tg2576 mice, treated or untreated with RSG. Animals were anesthetized with 2-2-2-tribromoethanol (20 mg/ml; Sigma, St. Louis, MO), followed by intracardiac perfusion with sucrose-based artificial cerebrospinal fluid (ACSF) consisting of the following (mM): 56 NaCl, 100 sucrose, 2.5 KCl, 20 glucose, 5 MgCl_2_, 1 CaCl_2_, 30 NaHCO_3_, and 1.25 NaH_2_PO_4_; osmolarity 300–310, pH 7.4. Brains were dissected and 250 μm horizontal hippocampal slices prepared with a vibratome VT1200 S (Leica, Buffalo Grove, IL) in iced, sucrose-based ACSF, continuously oxygenized and equilibrated to pH 7.4 with a mixture of 95% O_2_/5% CO_2_. Slices were then transferred to an incubation chamber with standard ACSF consisting of the following (in mM): 130 NaCl, 3.5 KCl, 10 glucose, 1.5 MgCl_2_, 1.4 CaCl_2_, 23 NaHCO_3_, and 1.25 NaH_2_PO_4_; osmolarity 300–310, oxygenated and equilibrated to pH 7.4 with a mixture of 95% O_2_/5% CO_2_ at 31°C. Before recordings, slices were equilibrated at room temperature in the recording chamber for 15–20 min.

#### Patch-clamp recording and data analysis.

**Fig. 1.**
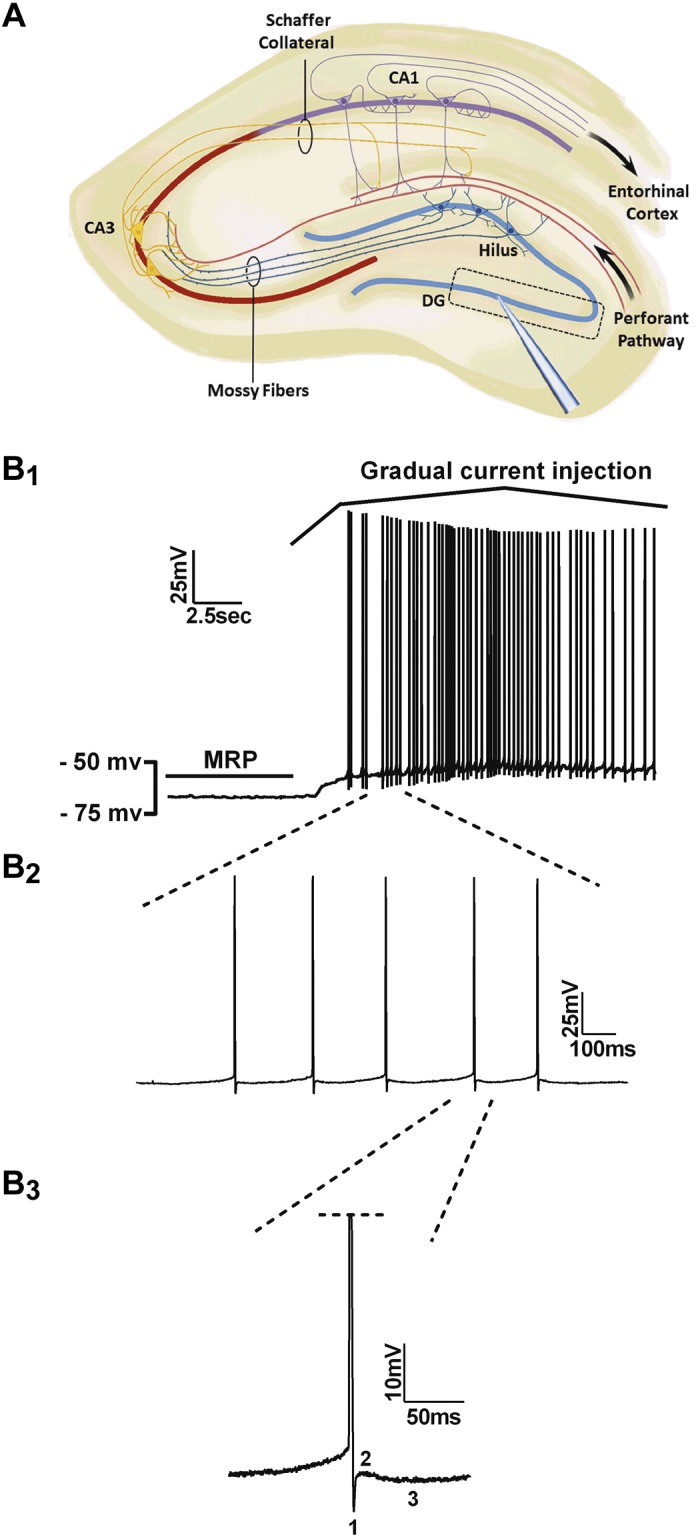
Selection and properties of visually identified neurons in the dentate gyrus (DG) circuit. *A*: schematic representation of the trisynaptic hippocampal circuit. The black box points to the general area (1st 3rd to the 2nd 3rd of the DG inferior blade) from which cells were chosen for whole-cell patch-clamp recordings. CA1 and CA3, *cornu ammonis* areas 1 and 3. *B*_*1*_: a typical DG granule cell at membrane resting potential (MRP) and after gradual current injection. *B*_*2*_: an inset of this trace showing spontaneous firing at higher resolution. *B*_*3*_: the characteristic triphasic nature of the after-hyperpolarization (AHP) phase in DG granule cells [1 corresponds to fast AHP (fAHP), 2 to after-spike depolarizing potential, and 3 to slow AHP (sAHP)].

After 1–2 h of recovery, brain slices were placed in a submerged recording chamber on the stage of an upright microscope (Axioskop 2 FS Plus; Zeiss, Thornwood, NY). Slices were perfused continuously at room temperature with standard ACSF (∼2 ml/min) in the presence of 20 μM bicuculline to block GABAergic synaptic transmission. Whole-cell patch-clamp recordings were obtained from visually identified DG granule cells from the first to second-third of the inferior blade of the DG using infrared differential interference contrast-infrared optics. Recording pipettes (4–7 MΩ) were fabricated from borosilicate glass (World Precision Instruments, Sarasota, FL), using a two-step vertical puller PC-10 (Narishige, Tokyo, Japan), and filled with intracellular solution containing the following (in mM): 120 K- hydroxymethanesulfinate, 10 KCl, 10 HEPES, 10 glucose, 2 MgCl_2_, 0.5 EGTA, 2 MgATP, and 0.5 Na_3_GTP; osmolarity 280–290, pH 7.3, adjusted with KOH. Whole-cell somatic recordings were performed using an Axopatch 200A amplifier (Molecular Devices, Sunnyvale, CA), low-pass filtered at 5 kHz, and sampled at 10–20 kHz using a Digidata 1200 analog-to-digital interface and pCLAMP 7 acquisition software (Molecular Devices). Seal formation and membrane rupture were done in voltage-clamp mode at a holding potential of −70 mV. After break-in, cells were maintained at a −70-mV holding potential in voltage-clamp mode for 1–2 min and then switched to current-clamp mode with a holding current of 0 pA to acquire membrane resting potential (MRP). Repetitive action-potential (AP) firing (Fig. 1) was evoked by gradual injection of a depolarizing current. To acquire input-output relationships and passive properties, all cells were then set to the membrane potential of −70 mV with injection of holding current (∼−25 to ∼+25 pA). Only cells showing a MRP more negative than −50 mV or a holding current between −25 and +25 pA at a membrane potential of −70 mV and stable access resistance (average series resistance was 22.4 ± 0.75 MΩ) were used for the subsequent analysis.

#### Patch-clamp data analysis.

A series of 500 ms current pulses (from −20 to +200 pA, 10 pA increments) was elicited to obtain AP firing trains. The AP current threshold (I_trh_) was defined as the current step at which at least one spike was induced. The AP voltage threshold (V_trh_) was defined as the voltage at which the first-time derivative of the rising phase of the AP exceeded 10 mV/ms. The first spike latency was defined as a time interval from the beginning of the current step to the first appeared spike. V_trh_ and first spike latency were measured at the I_trh _value. Input-output relationships were plotted either as number of spikes against current step or averaged instantaneous firing frequency against current step. Only spikes with overshoot were taken into analysis. Passive membrane properties, such as input resistance (R_in_) and membrane time constant (τ), were measured with current-clamp recordings from a membrane potential of −70 mV. For determination of R_in_, the steady-state values of the voltage responses to a series of current steps from −120 to +20 pA with 20 pA increments/step and a duration of 200 ms were plotted as a voltage-current relationship. R_in_ was calculated as the slope of the data points fitted with linear regression. Membrane τ was calculated by fitting a single exponential function to the first 100–150 ms at a −40-pA hyperpolarizing, 200-ms current step. The triphasic after-hyperpolarization (AHP) was analyzed from AP, induced by gradual injection of a depolarizing current (Fig. 1). Fast AHP (fAHP) was measured as the difference between the AP V_trh_ and the most negative after-spike potential; the spike ADP amplitude was measured as the difference between the peak amplitude of the fAHP and the most positive membrane potential following fAHP; slow AHP (sAHP) was measured as a difference between the AP V_trh_ and the most negative membrane potential after the spike (60–120 ms). All data were analyzed with pCLAMP 9 (Molecular Devices) and GraphPad Prism 6 software (GraphPad Software, San Diego, CA).

#### Statistical analysis.

**Fig. 2.**
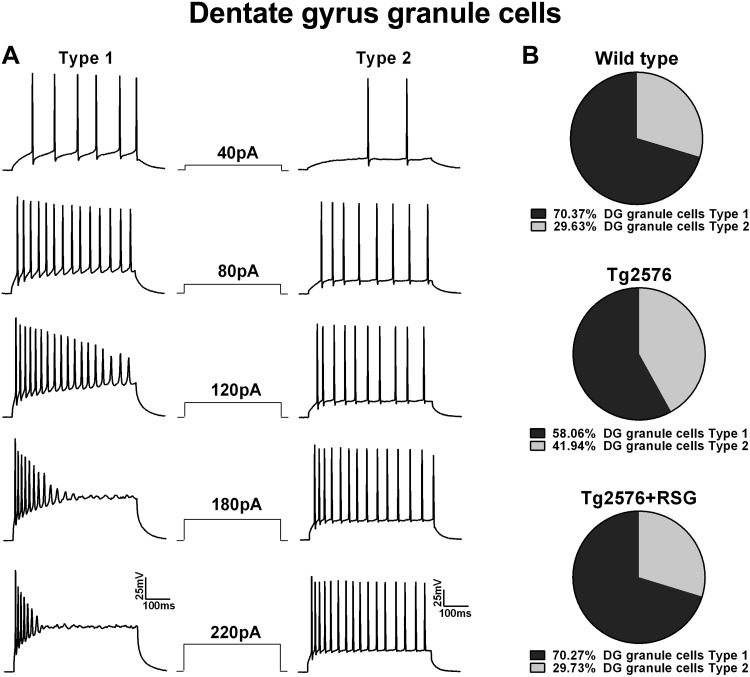
DG granule cells are characterized as type I and type II based on firing properties. *A*: DG granule cells sorted into type I and type II based on firing properties. Trains of action potentials (APs) were evoked by long, depolarizing current steps (500 ms). *B*: distribution of DG type I and type II neurons in wild-type, Tg2576, and rosiglitazone (RSG)-treated Tg2576 hippocampus. Type I cells are significantly more abundant in wild-type (∼70%) compared with type II (∼30%; *P* < 0.019, *Z* = 2.1, 1-proportion *Z*-test), but the difference between the 2 proportions is not statistically significant and reaches ∼50% in Tg2576 animals (*P* = 0.186, *Z* = 0.89, 1-proportion *Z*-test). Peroxisome proliferator-activated receptor-gamma (PPARγ) agonism normalizes the ratio of type I vs. type II cells (∼70% vs. 30%; *P* < 0.007, *Z* = 2.43, 1-proportion *Z*-test). RSG treatment does not affect the proportion of type I (*n* = 16, 66.7%) and type II (*n* = 8, 33.3%) in wild-type animals (*P* = 0.047, *Z* = 1.67, 1-proportion *Z*-test).

Data were analyzed with Student's *t*-test, one-way ANOVA, or Kruskal-Wallis test; then, all values were compared with the control with either post hoc Dunnett or Fisher least significant difference (LSD) test or post hoc Dunn test. The post hoc Dunnett test was the first choice for multiple comparisons, but in some cases, the less-stringent Fisher LSD test was applied to maximize the likelihood of identifying differences between experimental groups. The post hoc Dunn test was applied for multiple comparisons of samples that were not normally distributed. One-proportion *Z*-test was used for proportion analysis (Fig. 2). Data are presented as mean ± SE.

## RESULTS

In previous studies, we demonstrated that the synaptic properties of mPP inputs to DG granule neurons in 9-mo-old Tg2576 AD mice were altered due to presynaptic mechanisms and restored to normal function by 1-mo treatment with the PPARγ agonist RSG (Nenov et al. 2014). In the present study, we asked whether intrinsic firing properties of DG neurons are affected by Aβ pathology or if they are sensitive to RSG treatment. To gain insight into the intrinsic properties of DG granule neurons in this AD model, we applied whole-cell patch-clamp electrophysiology to DG granule neurons in acute hippocampal slices prepared from 9-mo-old wild-type and Tg2576 littermates, untreated or treated with RSG for 1 mo. Visually identified DG granule cells were selected from the first to second-third of the inferior blade (Fig. 1*A*). Current-clamp recordings were obtained from wild-type mice (*n* = 18 animals, *n* = 31 cells), Tg2576 mice (*n* = 17 animals, *n* = 33 cells), Tg2576 mice treated with RSG (*n* = 21 animals, *n* = 39 cells), and wild-type mice treated with RSG (*n* = 13 animals, *n* = 27 cells). Included in the analysis were cells that showed no spontaneous firing at the MRP (more negative than −50 mV) and/or exhibited a triphasic AHP (Fig. 1*B*) and a degree of firing adaptation (Fig. 2*A*). These criteria were used to exclude unhealthy cells or interneurons from the analysis (Scharfman 1992).

To examine the intrinsic firing properties of DG granule neurons, prolonged current step injections (500 ms) of 10 pA increments, from −20 pA up to 220 pA, were applied to evoke trains of APs. We found that the vast majority of cells in the four experimental conditions (wild-type, RSG-treated wild-type, Tg2576, and RSG-treated Tg2576) could be segregated into two distinct categories, which we termed type I and type II, based on firing properties resulting from these current steps (see details on cell-type distribution per animal in Table 1). Only 11 out of 130 total cells did not fall into either category and were excluded from the analysis.

**Table 1.** Number and cell type of dentate granule neurons recorded per animal for each experimental condition

Wild-type				Tg2576				Tg2576 + RSG				Wild-type + RSG			
Animal #	# of Cells (Cell Type)			Animal #	# of Cells (Cell Type)			Animal #	# of Cells (Cell Type)			Animal #	# of Cells (Cell Type)		
1	1 (t1)			1	1 (t1)			1	2 (t1, t1)			1	1 (t1)		
2	1 (t1)			2	1 (t2)			2	2 (t2, t2)			2	1 (t1)		
3	1 (t1)			3	3 (t1, t1, t2)			3	1 (t1)			3	2 (t2, t2)		
4	2 (t1, t1)			4	2 (t1, t2)			4	1 (t2)			4	2 (t1, t1)		
5	2 (t1, t1)			5	1 (t2)			5	2 (t1, t1)			5	2 (t1, t1)		
6	2 (t1, t2)			6	3 (t1, t1, t1)			6	1 (t1)			6	2 (t2, t1)		
7	1 (t2)			7	3 (t2, t2, t1)			7	1 (t2)			7	2 (t1, t1)		
8	1 (t2)			8	2 (t2, t1)			8	3 (t1, t2, t2)			8	1 (t1)		
9	3 (t2, t1, t1)			9	4 (t1, t1, t1, t1)			9	2 (t1, t1)			9	3 (t1, t2, t1)		
10	2 (t2, t2)			10	2 (t2, t2)			10	3 (t1, t1, t1)			10	6 (t2, t1, t2, t2, t2, t1)		
11	1 (t1)			11	1 (t1)			11	2 (t1, t2)			11	1 (t1)		
12	1 (t1)			12	1 (t2)			12	2 (t1, t2)			12	1 (t1)		
13	2 (t1, t1)			13	1 (t1)			13	1 (t2)						
14	5 (t2, t2, t1, t1, t1)			14	2 (t2, t2)			14	1 (t1)						
15	2 (t1, t1)			15	3 (t1, t1, t1)			15	3 (t1, t1, t1)						
				16	1 (t2)			16	2 (t1, t1)						
								17	1 (t1)						
								18	2 (t1, t1)						
								19	2 (t1, t2)						
								20	2 (t2, t1)						
								21	1 (t1)						
	# of Cells/Animal				# of Cells/Animal				# of Cells/Animal				# of Cells/Animal		
	Min	Max	Med		Min	Max	Med		Min	Max	Med		Min	Max	Med
	1	5	2		1	4	2		1	3	2		1	6	2

RSG, rosiglitazone; t1, dentate granule neuron type I; t2, dentate granule neuron type II; Min, minimum; Max, maximum; Med, median.

Type I cells reached a maximum number of spikes with current steps of moderate intensity and exhibited APs that eventually failed in the latter part of the train in response to current steps of high intensity (Fig. 2*A*). At current injections of high intensity (>150 pA), type I cells lacked sustained firing, and APs became progressively smaller in amplitude, eventually ceasing before the end of the current pulse. Unlike type I cells, type II cells fired throughout the entire range of current steps and exhibited firing rates proportional to the amplitude of the injected current. In type II cells, firing was sustained at all amplitudes without failure to generate APs, and the maximum number of spikes was observed at maximum stimulation intensities (Fig. 2*A*). In all four experimental groups, the distinction between type I and type II cell firing was made first at a moderate current step intensity of 90 pA that resulted in a higher number of APs in type I compared with type II (in wild-type, the number of AP was 14.1 ± 1.3 in type I vs. 8.8 ± 0.9 in type II; *P* = 0.0032). Then, at the maximum current step of 220 pA, firing was limited in type I due to AP failure throughout most of the train or sustained in type II cells (i.e., in wild-type, the number of AP was 5.4 ± 0.8 for type I vs. 17.3 ± 1.5 for type II; *P* = 0.00084, Student's *t*-test). We also found that the firing modality in the two cell types was mirrored by different values of R_in_ and I_trh_ throughout all four animal groups (Table 2). In wild-type mice, the R_in_ of type I cells was significantly higher compared with type II (443.3 ± 19.9 MΩ, *n* = 19, for type I vs. 297.2 ± 13.6 MΩ, *n* = 8, for type II; *P* = 0.000003, Student's *t*-test), whereas I_trh_ was significantly lower in type I compared with type II (25.3 ± 2.7 pA, *n* = 19, for type I and 41.3 ± 5.5, *n* = 8, for type II; *P* = 0.025, Student's *t*-test). In Tg2576 mice, untreated or treated with RSG, and wild-type treated with RSG, R_in_ of type I cells was significantly higher, and I_trh_ was typically lower compared with type II cells.

**Table 2.** Comparison between the number of action potentials, input resistance (R_in_), and current threshold (I_trh_) in type I and type II DG granule cells

	Wild-type			Tg2576			Tg2576 + RSG			Wild-type + RSG		
	Type I, *n* = 19	Type II, *n* = 8	*P* <	Type I, *n* = 18	Type II, *n* = 13	*P* <	Type I, *n* = 26	Type II, *n* = 11	*P* <	Type I, *n* = 16	Type II, *n* = 8	*P* <
# of APs at 90 pA current step	14.1 ± 1.3	8.8 ± 0.9	0.005	13.4 ± 0.8	10.6 ± 0.9	0.05	14.5 ± 0.8	8.4 ± 0.6	0.005	14.25 ± 0.8	7.9 ± 1.1	0.005
# of APs at 220 pA current step	5.4 ± 0.8	17.3 ± 1.5	0.005	7.3 ± 0.9	21.2 ± 0.9	0.005	7 ± 0.9	17.9 ± 0.8	0.005	7.1 ± 0.9	16.5 ± 1.2	0.005
R_in_, MΩ	443.3 ± 19.9	297.2 ± 13.6	0.005	419.1 ± 16.4	345.7 ± 14.9	0.005	440.6 ± 11.7	342.2 ± 22.4	0.005	444.5 ± 16.5	377.3 ± 12.9	0.005
I_trh_, pA	25.3 ± 2.7	41.3 ± 5.5	0.05	30.6 ± 2.7	36.9 ± 4.3	0.2	26.9 ± 1.9	41.8 ± 2.9	0.01	30 ± 2	42.5 ± 6.5	0.05

Type I cells were compared with type II cells for wild-type, RSG-treated wild-type, Tg2576, or RSG-treated Tg2576. Data are mean ± SE. *P* values obtained with Student's *t*-test.

DG, dentate gyrus; APs, action potentials.

Adult DG granule cells are comprised of a diverse population of newly born and mature neurons that develop and dynamically integrate in the DG circuit (Aimone et al. 2011; Nakashiba et al. 2012; Sahay et al. 2012). The intrinsic firing properties and ionic conductances in granule cells are thought to reflect their developmental stage and maturation level (Ge et al. 2007; Mongiat et al. 2009; Schmidt-Hieber et al. 2004; van Praag et al. 2002). Among DG granule cells, R_in_, I_trh_, and firing patterns have been used as signatures of the degree of maturation and circuitry integration. Our classification aligns with previous studies, with type I cells corresponding to a less mature phenotype, whereas the type II cells correlate with a more mature cell type, as described in Mongiat et al. (2009), Spampanato et al. (2012), and van Praag et al. (2002). Notably, the proportion of type I vs. type II cells was 70.4% (*n* = 19) and 29.6% (*n* = 8) in wild-type animals but switched to 58.1% (type I, *n* = 18) vs. 41.9% (type II, *n* = 13) in Tg2576, suggesting a decrement in proliferation, survival, maturation, or integration of DG granule neurons in the AD mouse. In RSG-treated Tg2576, the proportion of Tg2576 type I (70.3%, *n* = 26) and type II (29.7%, *n* = 11) cells was similar to the wild-type population distribution (Fig. 2*B*), suggesting a role of PPARγ signaling in controlling DG granule neuron heterogeneity. The proportion for type I and type II in RSG-treated wild-type was similar to wild-type (*n* = 16, 66.7% in type I, and *n* = 8, 33.3% in type II).

### 

#### Electrophysiological properties of type I granule cells in wild-type and Tg2576 mice.

**Fig. 3.**
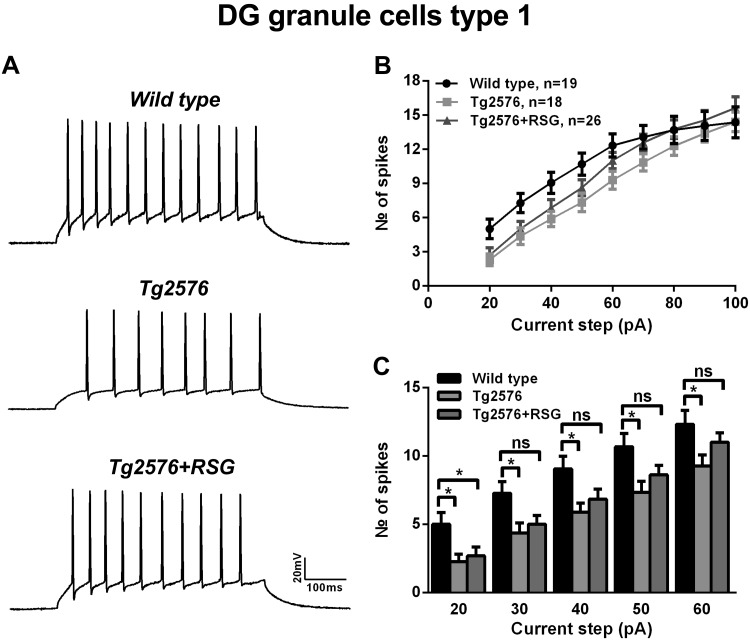
Characterization of firing properties of type I cells in Tg2576 and RSG-treated Tg2576 mice. *A*: representative traces of trains of APs induced by 100 pA depolarizing steps in wild-type (*top*), Tg2576 (*middle*), and RSG-treated Tg2576 (*bottom*) mice. *B* and *C*: input-output curves and histogram showing number of spikes elicited in response to 500 ms-long depolarizing current steps with 10 pA increments. The number of spikes/pulse was significantly lower in Tg2576 and normalized with RSG treatment compared with wild-type. For all experiments, results represent mean ± SE, *n* = 19 (wild-type), *n* = 18 (Tg2576), and *n* = 26 (Tg2576 + RSG). *P < 0.05, 1-way ANOVA, Dunnett post hoc test.

Alterations in firing properties induced by Aβ pathology have been described for hippocampal neurons (Brown et al. 2011; Santos et al. 2010; Yun et al. 2006). However, an elucidation of intrinsic and passive properties for distinct cell subtypes in the DG of an AD amyloidosis model, as well as following cognitive enhancement with PPARγ agonism, remains elusive. Thus we sought to examine these properties of type I and type II cells. Representative current-clamp traces of type I cell AP firing in wild-type and Tg2576, with and without RSG treatment, are shown in Fig. 3*A*. Repetitive firing was evoked by 500 ms-long current step at 100 pA. Type I cells from Tg2576 fired fewer spikes compared with wild-type, and RSG normalized the phenotype back to wild-type (Fig. 3*A*). Analysis of input-output curves revealed that the number of spikes elicited by current steps of low-to-moderate intensity (30–60 pA) was significantly lower in Tg2576 compared with wild-type mice and that RSG treatment restored the firing to levels not significantly different from control (Fig. 3*B*). The most pronounced phenotype in Tg2576 and its normalization with RSG was observed at a moderate current step (60 pA) with type I cells firing, on average, 12.3 ± 1 APs in wild-type DG (*n* = 19 cells), 9.3 ± 0.8 (*n* = 18 cells) in Tg2576, and 11 ± 0.7 (*n* = 26 cells) in Tg2576 + RSG (Fig. 3*C*; *P* < 0.05, one-way ANOVA, post hoc Dunnett test).

To gain information on conductances that might underlie the observed phenotype, the first spike latency, the AP I_trh_ and V_trh_ were examined, as shown in Table 3. The first spike latency was 159.2 ± 21 ms (*n* = 19 cells) in wild-type mice and was increased significantly in Tg2576 animals (270.8 ± 23.2 ms, *n* = 18 cells), but RSG treatment did not rescue the phenotype back to control (273.9 ± 25.2 ms, *n* = 26 cells; *P* < 0.01 one-way ANOVA, post hoc Dunnett test). Although a trend in increase of I_trh _and corresponding RSG rescue was observed, none of these changes were statistically different from one another (*P* > 0.05, one-way ANOVA, post hoc Dunnett test). The I_trh _was 25.3 ± 2.7 pA in wild-type mice (*n* = 19 cells), 30.6 ± 2.7 pA in Tg2576 mice (*n* = 18 cells), and 26.9 ± 1.9 pA in RSG-treated Tg2576 mice (*n* = 26 cells). Analysis with post hoc Fisher test indicated that V_trh_ was increased in Tg2576 mice (−43.9 ± 1.9 mV, *n* = 18 cells; *P* < 0.05) and rescued in RSG-treated Tg2576 mice (−45.8 ± 1.2 mV, *n* = 26 cells) compared with the wild-type control (−48.6 ± 1.1 mV, *n* = 19 cells; *P* < 0.05 post hoc Fisher test).

**Table 3.** Comparison of active and passive properties of type I DG granule cells under different experimental conditions

	Wild-type, *n* = 19	Tg2576, *n* = 18	Tg2576 + RSG, *n* = 26	*P*
First peak latency, ms	159.2 ± 21	270.8 ± 23.2	273.9 ± 25.2	0.002*
I_trh_, pA	25.3 ± 2.7	30.6 ± 2.7	26.9 ± 2	0.3*
V_trh_, mV	−48.6 ± 1.1	−43.9 ± 2	−45.8 ± 1.2	0.03^†^
MRP, mV	−59.6 ± 1.4	−66.6 ± 1.6	−64.7 ± 1.6	0.01*
R_in_, MΩ	443.3 ± 19.9	419.1 ± 16.4	440.6 ± 11.7	0.5*
τ, ms	26.1 ± 1.5	32.6 ± 2.3	28.5 ± 1.6	0.02^‡^
C_m_, pF	62.3 ± 5.8	79.3 ± 5.6	66.9 ± 4.3	0.03^†^

Tg2576- and RSG-treated Tg2576 were compared with wild-type. Data are mean ± SE.

* *P* values obtained with 1-way ANOVA and post hoc Dunnett test;

† *P* values were obtained with post hoc Fisher test; and

‡ *P* values obtained with post hoc Dunnett test.

V_trh_, voltage threshold; MRP, membrane resting potential; τ, time constant; C_m_, membrane capacitance.

**Fig. 4.**
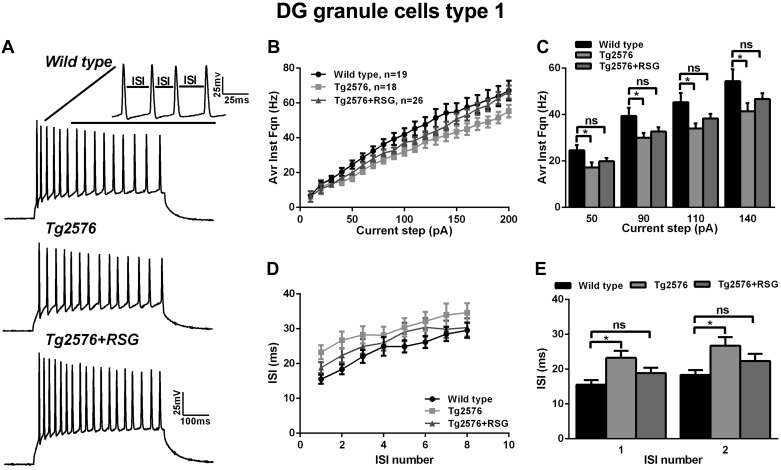
Instantaneous firing frequency in type I neurons is decreased in Tg2576 animals and rescued by RSG. *A*: representative traces of trains of APs induced by 110 pA depolarizing steps in wild-type (*top*), Tg2576 (*middle*), and Tg2576 + RSG (*bottom*) mice. *B* and *C*: input-output curves and histogram showing average instantaneous firing frequency (Avr Inst Fqn) derived from trains of APs elicited in response to 500 ms-long depolarizing current steps with 10 pA increments. The average instantaneous firing frequency for steps between 90 pA and 140 pA was significantly lower in Tg2576 animals compared with wild-type and was normalized by RSG treatment (*P < 0.05, 1-way ANOVA, Dunnett post hoc test). *D* and *E*: interspike intervals (ISIs) plotted as a function of ISI number (*D*) and summary histogram (*E*), showing increased ISI in Tg2576 type I neurons normalized to wild-type control through the PPARγ agonism. The most pronounced effect of amyloid-β (Aβ) pathology and RSG rescue on ISI was observed at the beginning of the AP train (ISI *numbers 1* and *2*). *P < 0.05, 1-way ANOVA with Dunnett and Fisher post hoc tests. Results represent mean ± SE; *n* = 19 (wild-type), *n* = 18 (Tg2576), *n* = 26 (Tg2576 + RSG).

To understand better the firing pattern and membrane properties of type I cells, we examined average instantaneous firing frequency, interspike intervals (ISIs), triphasic AHP, MRP, R_in_, membrane capacitance (C_m_), and τ across the three experimental groups. Type I cells exhibited a reduced average instantaneous firing frequency and increased ISI in Tg2576 compared with wild-type, and RSG treatment normalized the phenotype (Fig. 4). Input-output curves over a wide range of current steps (10–200 pA) showed a significant reduction in the average instantaneous firing frequency of type I cells at current steps of moderate intensity (50–140 pA) in Tg2576 compared with wild-type littermates (at 140 pA: 54.4 ± 5.1 Hz, *n* = 19 cells, in wild-type vs. 41.4 ± 3.5 Hz, *n* = 18 cells, in Tg2576; *P* < 0.05, one-way ANOVA with post hoc Dunnett test; Fig. 4, *B* and *C*). These changes were accompanied by a significant increase in the ISI observed, especially at the beginning of the AP train at the 110-pA current step (15.5 ± 1.3 ms, *n* = 19 cells, in wild-type vs. 23.3 ± 2 ms, *n* = 18 cells, in Tg2576; *P* < 0.05, one-way ANOVA with post hoc Dunnett test; Fig. 4, *D* and *E*). Importantly, RSG treatment rescued both instantaneous firing frequency and ISI, bringing these values back to wild-type (Fig. 4, *C–E*; *P* > 0.05, one-way ANOVA, post hoc Dunnett test).

**Fig. 5.**
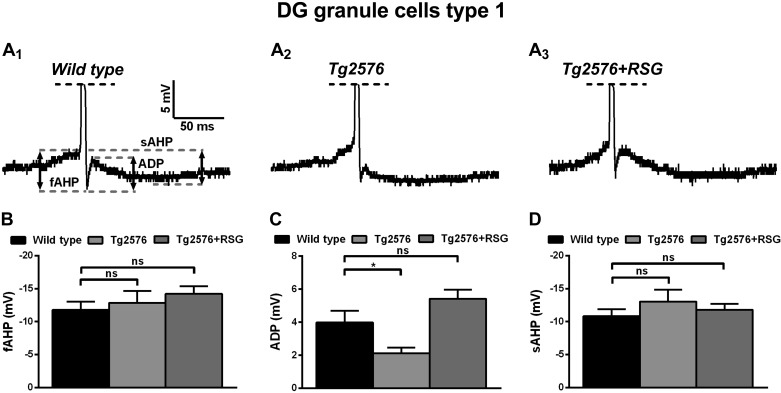
AP triphasic AHP of type I granule cells in wild-type, Tg2576, and RSG-treated Tg2576 animals. *A*: representative traces of triphasic AHP in wild-type (*A*_*1*_), Tg2576 (*A*_*2*_), and RSG-treated Tg2576 (*A*_*3*_). Dashed lines indicate measured levels of fAHP, spike after-depolarization (ADP), and sAHP. *B*: fAHP did not vary across genetic or treatment conditions (*P* > 0.05, 1-way ANOVA, post hoc Dunnett). *C*: ADP was significantly lower in Tg2576 compared with wild-type control and rescued by RSG treatment (*P < 0.05 Kruskal-Wallis test, post hoc Dunn). *D*: sAHP did not vary across conditions (*P* > 0.05 Kruskal-Wallis test, post hoc Dunn). Results represent mean ± SE; *n* = 18 (wild-type), *n* = 18 (Tg2576), *n* = 24 (Tg2576 + RSG).

Analysis of the AP triphasic AHP properties revealed that the spike ADP was decreased significantly in type I cells in Tg2576 animals (2.1 ± 0.3 mV, *n* = 18 cells) compared with wild-type (3.9 ± 0.7 mV, *n* = 18; *P* < 0.05 Kruskal-Wallis test, post hoc Dunn test) and restored to normal values by RSG (5.4 ± 0.6 mV, *n* = 24 cells; Fig. 5, *A* and *C*). Other spike potentials preceding ADP (fAHP, −11.8 ± 1.2 mV, *n* = 18 cells for wild-type; −12.8 ± 1.8 mV, *n* = 18 cells for Tg2576; −14.2 ± 1.2, *n* = 24 cells for RSG-treated Tg2576; *P* > 0.05, one-way ANOVA, post hoc Dunnett) and following ADP (sAHP, −10.8 ± 1.1 mV, *n* = 18 cells for wild-type; −13 ± 1.8 mV, *n* = 18 cells for Tg2576; −11.8 ± 0.9 mV, *n* = 24 cells for RSG-treated Tg2576; *P* > 0.05, Kruskal-Wallis, post hoc Dunn test) were unchanged across conditions (Fig. 5, *B* and *D*).

When we examined the MRP (Table 3), Tg2576 type I cells displayed more negative values relative to wild-type (−59.6 ± 1.4 mV, *n* = 17 cells, in wild-type vs. −66.6 ± 1.6 mV, *n* = 18 cells in Tg2576; *P* < 0.01 one-way ANOVA, post hoc Dunnett), a response unaffected by RSG (−64.7 ± 1.6 mV, *n* = 26 cells in RSG-treated Tg2576; *P* < 0.05, one-way ANOVA, post hoc Dunnett). In type I cells, R_in_ from the responses to hyperpolarizing steps was similar among the three groups (*P* > 0.05, one-way ANOVA). In contrast, τ was increased in Tg2576 cells compared with wild-type (32.6 ± 2.3 ms, *n* = 18, in Tg2576 vs. 26.05 ± 1.5 ms, *n* = 19, in control; *P* < 0.05 with post hoc Dunnett test), and RSG treatment normalized this property (28.5 ± 1.6 ms, *n* = 26; *P* > 0.05, one-way ANOVA). Furthermore, analysis with post hoc Fisher test indicated that C_m_ was increased in Tg2576 cells (79.3 ± 5.6 pF, *n* = 18) compared with wild-type (62.3 ± 5.8 pF, *n* = 19; *P* < 0.05 with post hoc Fisher test), and RSG treatment normalized C_m_ to wild-type values (66.9 ± 4.3 pF, *n* = 26; *P* > 0.05, one-way ANOVA).

Finally, the effect of RSG on active and passive properties of type I cells in wild-type animals was examined (Table 4). RSG caused significant changes in intrinsic firing properties and V_trh_, with no effects on other parameters.

**Table 4.** Comparison of firing, active, and passive properties of type I DG granule cells between wild-type and RSG-treated wild-type

DG GC Type I	Wild-type, *n* = 19	Wild-type + RSG, *n* = 16	*P*
MRP, mV	−59.6 ± 1.4	−61.9 ± 1.7	0.3
V_trh_, mV	−48.6 ± 1.1	−44.9 ± 1.1	0.03*
I_trh_, mV	25.3 ± 2.7	30 ± 2	0.18
C_m_, pF	62.3 ± 5.8	67.6 ± 3.6	0.46
R_in_, MΩ	443.3 ± 19.9	444.5 ± 16.5	0.96
τ, ms	26.05 ± 1.5	29.9 ± 1.8	0.1
fAHP, mV	−11.8 ± 1.2	−12.4 ± 1.4	0.74
ADP, mV	3.9 ± 0.7	4.6 ± 0.6	0.47
sAHP, mV	−10.8 ± 1.05	−11.1 ± 1.1	0.85
Number of APs at Current Step of			
20, pA	5 ± 0.9	3.3 ± 1.2	0.31
30, pA	7.3 ± 0.8	3.6 ± 0.6	0.002*
40, pA	9.05 ± 0.9	5.9 ± 0.7	0.01*
50, pA	10.7 ± 0.9	8.2 ± 0.7	0.04*
60, pA	12.3 ± 1.02	10.2 ± 0.7	0.09
Instantaneous Firing Frequency (Hz) at Current Step of			
50, pA	24.6 ± 2.3	18.1 ± 1.3	0.02*
90, pA	39.4 ± 3.5	29.9 ± 1.6	0.02*
110, pA	45.3 ± 3.9	34.6 ± 1.8	0.02*
140, pA	54.4 ± 5.1	40.9 ± 2.2	0.02*

Data are mean ± SE.

* *P* values obtained with Student's *t*-test.

GC, granule cell; fAHP, fast after-hyperpolarization; ADP, after-depolarization; sAHP, slow AHP.

Collectively, these data show that compared with wild-type littermates, Tg2576 DG type I cells exhibit decreased intrinsic excitability and ADP accompanied by increased τ and C_m_, which are normalized by RSG treatment. These changes in active and passive membrane properties might be indicative of complex alterations of functional connectivity and potentially, morphological properties of type I cells in response to Aβ amyloidosis, most of which are restored to statistically normal values through PPARγ agonism.

#### Electrophysiological properties of type II granule cells in wild-type and Tg2576 mice.

**Fig. 6.**
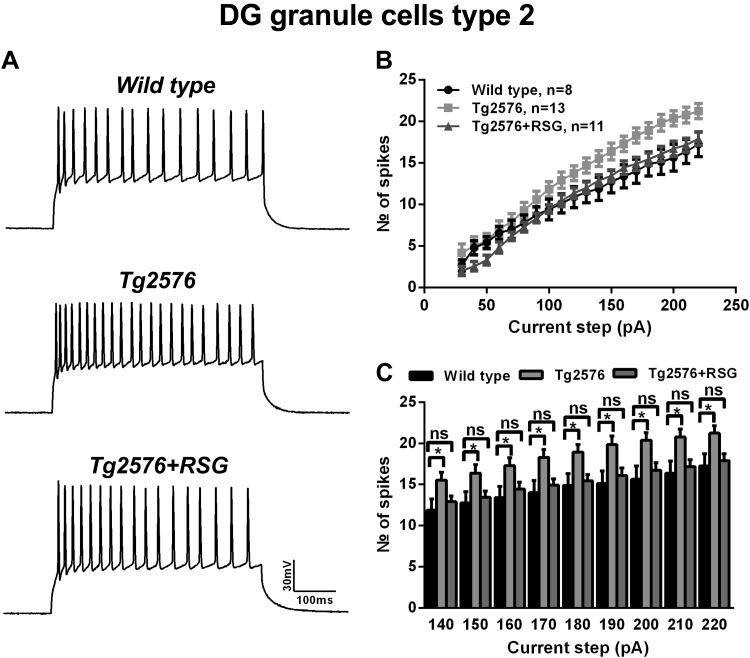
Characterization of firing properties of type II cells in Tg2576 and RSG-treated Tg2576 mice. *A*: representative traces of trains of APs induced by 200 pA depolarizing steps in wild-type (*top*), Tg2576 (*middle*), and RSG-treated Tg2576 (*bottom*) mice. *B* and *C*: input-output curves and graph bars showing number of spikes elicited in response to 500 ms-long depolarizing current steps with 10 pA increments. The number of spikes/pulse was significantly higher in Tg2576 and rescued with RSG treatment compared with wild-type. Results represent mean ± SE; *n* = 8 (wild-type), *n* = 13 (Tg2576), *n* = 11 (Tg2576 + RSG). *P < 0.05, 1-way ANOVA, Dunnett post hoc test.

The same current-clamp protocols used to characterize type I cells were applied to study intrinsic firing and membrane properties of type II neurons in response to Aβ pathology, with or without subsequent PPARγ agonism with RSG. Representative AP traces show that in contrast to type I, type II neurons in Tg2576 animals were more excitable than wild-type (Fig. 6*A*). Tg2576 type II cells exhibited sustained, repetitive firing in response to moderate-to-high depolarization steps with a higher number of spikes than wild-type. RSG treatment normalized the excitability of Tg2576 type II cells to wild-type, as evidenced by input-output curves (Fig. 6*B*). These responses were even more apparent at moderate and high current steps (Fig. 6*C*; *P* < 0.05, one-way ANOVA with post hoc Dunnett test). At maximal current injections (220 pA current step), type II cells fired, on average, 17.25 ± 1.5 spikes in wild-type (*n* = 8), 21.2 ± 0.9 in Tg2576 (*n* = 13), and 17.9 ± 0.8 in Tg2576 animals treated with RSG (*n* = 11), with the latter indistinguishable from wild-type (Fig. 6, *B* and *C*; *P* < 0.05, one-way ANOVA with post hoc Dunnett test). Additional measurements revealed that first-peak latency, I_trh_, nor V_trh_ was significantly different across the genotypes and treatment conditions (*P* > 0.05, one-way ANOVA, post hoc Dunnett test; Table 5).

**Table 5.** Comparison of active and passive properties of type II DG granule cells under different experimental conditions

	Wild-type (*n* = 8)	Tg2576 (*n* = 13)	Tg2576 + RSG (*n =* 11)	*P*
First peak latency, ms	265.3 ± 17.4	303.2 ± 26.8	296.5 ± 36	0.7
I_trh_, pA	41.3 ± 5.5	36.9 ± 4.3	41.8 ± 2.9	0.6
V_trh_, mV	−45.2 ± 2.7	−43.9 ± 1.9	−43 ± 1.9	0.8
MRP, mV	−69.2 ± 0.9	−72.9 ± 1.5	−67.1 ± 2.3	0.06
R_in_, MΩ	297.2 ± 13.6	345.7 ± 14.9	342.2 ± 22.4	0.16
τ, ms	31.9 ± 1.8	31.5 ± 2	30.7 ± 1.9	0.9
C_m_, pF	108.7 ± 9	89.1 ± 7.1	88.5 ± 6.3	0.16

Tg2576 and RSG-treated Tg2576 were compared with wild-type. Data are mean ± SE. *P* values were obtained with 1-way ANOVA and post hoc Dunnett test.

**Fig. 7.**
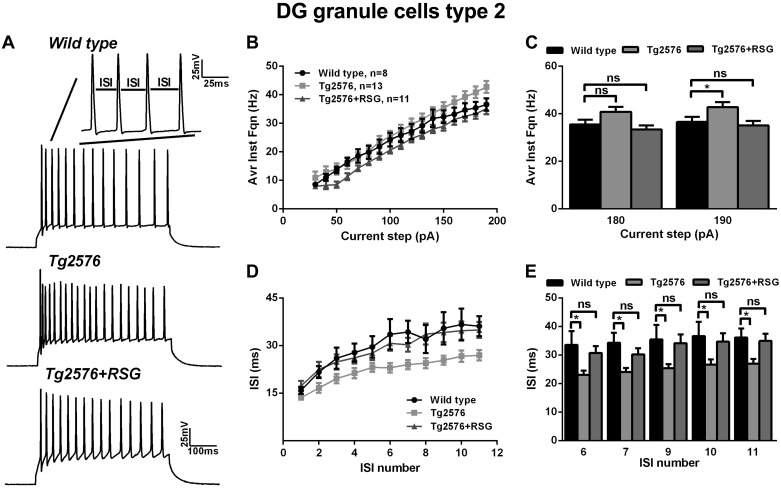
Instantaneous firing frequency in type II neurons is decreased in Tg2576 animals and rescued by RSG. *A*: representative traces of trains of APs induced by 190 pA depolarizing steps in wild-type (*top*), Tg2576 (*middle*), and Tg2576 + RSG (*bottom*) mice. *B* and *C*: input-output curves and graph bars showing average instantaneous firing frequency derived from trains of APs elicited in response to 500 ms-long depolarizing current steps with 10 pA increments. The average instantaneous firing frequency for steps between 90 pA and 140 pA was significantly higher in Tg2576 animals compared with wild-type and was rescued by RSG treatment (*P < 0.05, 1-way ANOVA, Dunnett post hoc test). *D* and *E*: ISIs plotted as a function of ISI number (*D*) and summary graph bar representation (*E*), showing reduced ISI in Tg2576 type I neurons rescued to the wild-type control by RSG treatment. The most pronounced effect of Aβ pathology and RSG rescue on ISI was observed at the beginning of the AP train (ISI *numbers 1* and *2*). *P < 0.05, 1-way ANOVA with Dunnett and Fisher post hoc tests. Results represent mean ± SE; *n* = 8 (wild-type), *n* = 13 (Tg2576), *n* = 11 (Tg2576 + RSG).

**Fig. 8.**
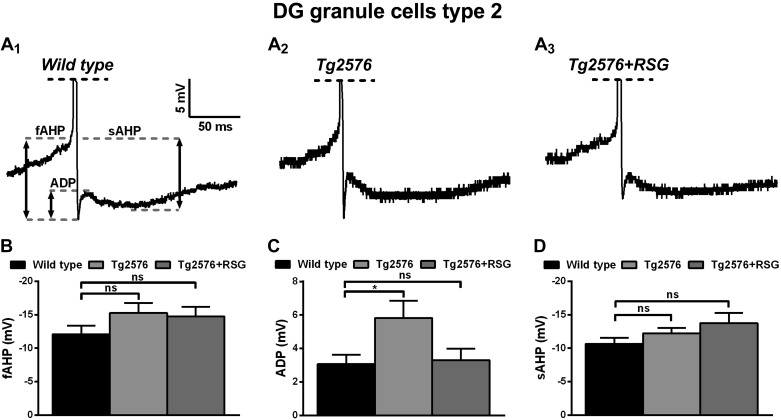
AP triphasic AHP of type II granule cells in wild-type, Tg2576, and RSG-treated Tg2576 animals. *A*: representative traces of triphasic AHP in wild-type (*A*_*1*_), Tg2576 (*A*_*2*_), and RSG-treated Tg2576 (*A*_*3*_). Dashed lines indicate measured levels of fAHP, spike ADP, and sAHP. *B*: fAHP did not vary across conditions (*P* > 0.05, 1-way ANOVA, post hoc Dunnett). *C*: ADP was significantly higher in Tg2576 compared with wild-type control and rescued by RSG treatment (*P < 0.05, 1-way ANOVA, post hoc Fisher). *D*: sAHP did not vary across conditions (*P* > 0.05, 1-way ANOVA, post hoc Dunnett). Results represent mean ± SE; *n* = 8 (wild-type), *n* = 12 (Tg2576), *n* = 10 (Tg2576 + RSG).

Evaluation of instantaneous firing frequency and ISI revealed additional signs of hyperexcitability of type II cells in Tg2576 animals and rescue upon RSG treatment. The increase in average instantaneous firing frequency with the magnitude of injected current was greater in Tg2576 type II cells compared with wild-type (Fig. 7, *A* and *B*), reaching significant differences for current steps of high intensity (Fig. 7*C*; 190 pA, 36.5 ± 2.2 Hz, *n* = 19 cells in wild-type; 42.7 ± 2.1 Hz, *n* = 18 in Tg2576; and 35.1 ± 1.9 Hz, *n* = 18 in Tg2576 + RSG; *P* < 0.05, one-way ANOVA with post hoc Dunnett test). This phenotype was accompanied by a decrease in ISI (at high current step injection, 190 pA), evident throughout the train, indicating a deficit in frequency adaptation (Fig. 7, *D* and *E*). RSG treatment rescued instantaneous firing frequency and ISI back to wild-type (Fig. 7; *P* < 0.05 one-way ANOVA with post hoc Dunnett or Fisher tests for selected train intervals). Interestingly, further analysis of the AP triphasic AHP revealed that ADP was increased significantly in type II cells in Tg2576 animals (5.8 ± 1 mV, *n* = 12 cells) compared with wild-type (3.1 ± 0.6 mV, *n* = 8 cells) and restored to normal values by RSG (3.3 ± 0.7, *n* = 10; *P* < 0.05 one-way ANOVA, post hoc Fisher; Fig. 8, *A* and *C*). fAHP (−12.1 ± 1.2 mV, *n* = 8 cells for wild-type; −15.3 ± 1.5 mV, *n* = 12 cells for Tg2576; −14.8 ± 1.4 mV, *n* = 10 cells for RSG-treated Tg2576; *P* > 0.05 one-way ANOVA, post hoc Dunnett) and sAHP (−10.7 ± 0.9 mV, *n* = 8 cells for wild cells; −12.2 ± 0.8 mV, *n* = 12 cells for Tg2576; −13.8 ± 1.5 mV, *n* = 10 cells for RSG-treated Tg2576; *P* > 0.05 one-way ANOVA, post hoc Dunnett) were unchanged across conditions (Fig. 8, *B* and *D*).

Finally, Table 5 shows that the MRP was −69.2 ± 0.9 mV in wild-type (*n* = 8 cells), and there was a trend to a hyperpolarizing shift of MRP in Tg2576 (−72.9 ± 1.5 mV, *n* = 13 cells), but none of these values were statistically different from one another (*P* = 0.06, one-way ANOVA with post hoc Dunnett test). Likewise, analysis of R_in_, τ, and C_m_ showed no statistical difference in τ among experimental conditions; there was a trend for increased R_in_ (345.7 ± 14.9 MΩ, *n* = 13 cells) and decreased C_m_ (89.1 ± 7.1 pF, *n* = 13 cells) in Tg2576 compared with wild-type (297.2 ± 13.6 MΩ and 108.7 ± 9 pF, *n* = 8 cells), but RSG treatment had no significant effect on these parameters (342.2 ± 22.4 MΩ and 88.5 ± 6.3, *n* = 11 cells; *P* > 0.05 one-way ANOVA with post hoc Dunnett test).

Further analysis of the RSG effect on wild-type type II cells showed an increase of R_in_ compared with the untreated wild-type, with no effect on any other parameters (Table 6). Thus in contrast to type I, type II cells in the AD mouse model are hyperactive, and RSG treatment normalizes their function back to wild-type.

**Table 6.** Comparison of firing, active, and passive properties of type II DG granule cells between wild-type and RSG-treated wild-type

DG GC Type II	Wild-type, *n* = 8	Wild-type + RSG, *n* = 8	*P*
MRP, mV	−69.2 ± 0.9	−72.6 ± 2.5	0.25
V_trh_, mV	−45.2 ± 2.7	−43 ± 2.5	0.56
I_trh_, mV	41.3 ± 5.5	42.5 ± 6.5	0.88
C_m_, pF	108.7 ± 9	112 ± 15.1	0.86
R_in_, MΩ	297.2 ± 13.6	377.3 ± 12.9	0.001*
τ, ms	31.9 ± 1.8	42.78 ± 6.4	0.14
fAHP, mV	−12.1 ± 1.2	−15.3 ± 2.1	0.21
ADP, mV	3.1 ± 0.6	3.3 ± 0.8	0.77
sAHP, mV	−10.7 ± 0.9	−14.4 ± 1.9	0.1
Number of APs at Current Step of			
140, pA	11.9 ± 1.4	11.8 ± 1.2	0.95
150, pA	12.8 ± 1.4	12.6 ± 1.2	0.95
160, pA	13.4 ± 1.4	12.5 ± 1.2	0.64
170, pA	14 ± 1.4	13.5 ± 1.3	0.8
180, pA	14.9 ± 1.4	13.8 ± 1.2	0.56
190, pA	15.1 ± 1.5	14.6 ± 1.3	0.81
200, pA	15.6 ± 1.6	15.5 ± 1.2	0.95
210, pA	16.4 ± 1.5	15.9 ± 1.3	0.8
220, pA	17.3 ± 1.5	16.5 ± 1.2	0.7
Instantaneous Firing Frequency (Hz) at Current Step of			
180, pA	35.4 ± 2.1	30.4 ± 2.4	0.13
190, pA	36.5 ± 2.2	31.7 ± 2.4	0.16

Data are mean ± SE.

* *P* values obtained with Student's *t*-test.

Overall, these data indicate clear mechanistic distinctions between type I and type II cells in normal and disease-model animals. Aβ pathology resulted in altered functional properties of these DG neurons, and PPARγ agonism restored most of these, suggesting that PPARγ signaling promotes a global program aiming at normalizing DG function during AD amyloidosis.

## DISCUSSION

The DG is the hippocampal gateway for new memory formation, and its functional and structural integrity is particularly susceptible to disruption in early AD. In some humans with early AD and in mouse models for AD-like amyloidosis, impaired memory can be improved with PPARγ activation (Risner et al. 2006; Rodriguez-Rivera et al. 2011; Watson et al. 2005). However, the cellular mechanisms underlying PPARγ cognitive enhancement are poorly understood. In previous studies, we showed that along with improving cognition through a brain-specific activity, RSG treatment rescues aberrant synaptic activity of mPP inputs to DG granule neurons in the Tg2576 animal models for AD (Nenov et al. 2014). In the present study, we applied whole-cell patch-clamp recordings to characterize resting and active membrane properties of DG granule neurons in the Tg2576 animal model and examined their sensitivity to RSG. Our findings complement and extend the repertoire of Aβ pathology-induced phenotypes in the DG circuit of AD animals and show that in addition to synaptic activity, PPARγ agonists restore the DG granule cell function by normalizing intrinsic properties. Thus PPARγ agonists may antagonize AD cognitive dysfunction by rescuing the DG output signals to the CA3 area, which are required for early memory formation.

Our present findings, supported by previous studies, allowed us to segregate granule neurons in the DG into two functionally distinct populations of cells with properties that align with previously described type I (less mature) and type II (more mature) phenotypes, based on R_in_, I_trh, _and firing patterns (Mongiat et al. 2009; Schmidt-Hieber et al. 2004; Spampanato et al. 2012; van Praag et al. 2002). In Tg2576 mice that model AD-like amyloidosis and cognitive impairment reminiscent of early AD (Rodriguez-Rivera et al. 2011), the proportion of these cells is skewed toward type II, away from the wild-type distribution of cell types. Furthermore, the intrinsic excitability of both cell types is aberrant in Tg2576: type I neurons fire significantly less APs and exhibit reduced instantaneous firing frequency, whereas type II neurons are more excitable and present with the opposite phenotypes. AD pathology is associated with loss in memory formation, functional alterations of DG circuitry, and propensity to epileptic burst activity (Born et al. 2014; Davis et al. 2014; Kamenetz et al. 2003). Thus the changes in type I vs. type II proportion and their relative aberrant firing abilities in Tg2576 observed in these studies provide a potential mechanism underlying the reported hyperactivity of the DG in AD (Born et al. 2014; Davis et al. 2014; Hazra et al. 2013; Palop et al. 2007; Ziburkus et al. 2013). The ratio of type I vs. type II neurons and their altered membrane properties are restored to normal function upon RSG treatment. These results further corroborate ours and others' prior studies revealing aberrant synaptic transmission in the entorhinal cortex-DG synaptic circuit and concomitant memory deficits in Tg2576 animals that are ameliorated by RSG-mediated PPARγ agonism (Denner et al. 2012; Escribano et al. 2010; Jahrling et al. 2014; Rodriguez-Rivera et al. 2011).

### 

#### Type I and type II granule cells: effects of PPARγ agonism.

We identified two types of neurons based on established electrophysiological properties characterized in previous studies that used passive and active properties to sort newly generated from mature granule neurons (van Praag et al. 2002; Yun et al. 2006). Our study does not provide an unequivocal marker-based sorting of type I and type II cells, and as such, it precludes a true assessment of the actual developmental stage of these cells and their presumptive level of plasticity and integration in the DG circuit. Nonetheless, our classification type I cells (which might correspond to a less mature phenotype) exhibit: *1*) a firing pattern characterized by high firing frequency in response to stimuli of moderate intensity and tendency to AP failure in the latter part of trains evoked by stimuli of high intensity (van Praag et al. 2002); *2*) high R_in_ and low I_trh_ (Liu et al. 1996, 2000; Mongiat et al. 2009); *3*) relative depolarized MRP; and *4*) low C_m_ and τ. Except for a higher R_in_, type I cells in our study align well with the newly generated cells described by van Praag et al. (2002). Conversely, type II neurons resembled more closely integrated DG granule cells that exhibit lower R_in_ and higher I_trh_, indicative of a complete repertoire of ion channels, as in fully mature neurons, and fire constantly throughout the train (Mongiat et al. 2009; Scharfman 1992; van Praag et al. 2002).

We found that the proportion of type I cells in wild-type animals is predominant, representing ∼70% of the population. In Tg2576, this ratio is disturbed to ∼58% and restored to “normal” proportions by RSG. These results are in line with the well-established link between disruption of proliferation, differentiation, and maturation of adult-born neurons and AD (Krezymon et al. 2013; Li et al. 2008; Shruster et al. 2010) and support a potential role for PPARγ in promoting granule cell maturation (Morales-Garcia et al. 2011; Wang et al. 2011). However, a puzzling result from our study is that the number of type I, presumably less mature cells, appears to exceed type II in wild-type animals. This counter-intuitive result requires further investigations, given that our wild-type animals are 9 mo old, and granule neurons at this age are unlikely to be predominantly immature and plastic.

The observed changes in neuronal excitability support the well-established role of the DG neuron excitability in early AD pathology (Cuevas et al. 2011; Hermann et al. 2009; Kamenetz et al. 2003; Park et al. 2013; Ripoli et al. 2013; Rowan et al. 2005). We found that not only the ratio of type I vs. type II cells is changed in Tg2576 animals, but also, neurons within each of these two categories exhibit altered firing compared with the same type in healthy animals. Maximum number of spikes and instantaneous frequency are decreased in type I yet are upregulated in type II neurons. In both cases, PPARγ agonism treatment restored firing modalities to wild-type levels. Some other parameters, such as first peak latency, MRP were altered in Tg2576, and insensitive to RSG, suggesting very distinct cell-specific repertoires of ion channel conductances that are targeted by the PPARγ axis.

The observed shift in membrane excitability properties associated with Tg2576 Aβ pathology in type I vs. type II cells is consistent with previous reports (Hazra et al. 2013; Palop et al. 2007; Sanchez et al. 2012; Ziburkus et al. 2013). Aβ-induced hyperactivity has been detected at the single-cell firing level and in epileptic-like bursts recorded in cortical and hippocampal neural networks of mouse models for AD-like amyloidosis (Busche et al. 2012; Palop et al. 2007). However, Aβ pathology has also been associated with decreased neuronal firing in the DG, prefrontal cortex, and the CA1 hippocampal region (Haghani et al. 2012; Kaczorowski et al. 2011; Yun et al. 2006). Our cell-type stratification of neuronal firing phenotypes emphasizes the complexity of factors that leads to hippocampal circuit disruption in AD and may be applied to other studies to help reconcile some contrasting results in the literature (Santos et al. 2010).

Although the criteria used to sort type I vs. type II cells were based on firing, R_in_, and I_trh_, additional passive and active membrane properties seemed to associate preferentially with one cell type vs. the other. For example, τ and C_m_ values in type I cells were significantly different from type II in the wild-type animals but were more homogenous in Tg2576 (Tables 3 and 5). Likewise, MRP and τ values segregated with type I and type II categories in wild-type and in Tg2576, but they did not differ in type I vs. type II upon RSG treatment (Tables 3 and 5). These intragroup (i.e., type I vs. type II) differences in passive and active membrane properties, other than R_in_, I_trh_, and firing, precluded a more rigorous separation of the two cell types, given the multivariate parameters across the experimental conditions. Further strata, representing intermediate developmental stages between a strict delineation of type I and type II groups or additional consideration of homeostatic compensatory mechanisms implicating ion channels (MRP) or cell architecture (τ and C_m_) might associate these divergent parameters more exquisitely with the AD state and the effects of PPARγ agonism.

#### Potential mechanisms underlying changes in neuronal excitability induced by RSG.

Type I and type II cells exhibit very distinct firing patterns that imply an underlying diversity of voltage-gated ion channels. In both cell types, Aβ pathology modifies the maximum number of spikes and instantaneous firing frequency with no effects on R_in_ and I_trh_, and these phenotypes are rescued by RSG. The action of RSG on Tg2576 neuronal firing is consistent with a long-term, PPARγ-dependent reprogramming of the transcriptomic and proteomic landscapes leading to Aβ clearance-mediated actions of PPARγ (Denner et al. 2012; Escribano et al. 2010).

Changes in the spike ADP in Tg2576 animals are found in both cell types, and in both cases, these changes are rescued by RSG treatment. In type I, ADP is decreased in Tg2576 and brought back to control values by RSG, whereas in type II cells, ADP is upregulated in Tg2576 and restored to a normal level by RSG. The bidirectional change of ADP amplitude and its RSG rescue correlate with the observed changes in neuronal firing for both type I and type II cells, providing a potential mechanistic model to explain the observed phenotypes. Moreover, our data are in line with another study, where aberrant, intrinsic excitability in AD animal model CA1 neurons was associated with alternation in the amplitude of ADP (Brown et al. 2011). Likely ion channel candidates underlying ADP are the Kv7/M-type potassium channels (KCNQ-M) and/or T-type Ca^2+^ channels (Chen and Yaari 2008; Park and Lee 2002; Zhang et al. 1993). Thus Aβ pathology and RSG rescue might modify ADP through opposite effects on voltage-dependent K^+^ and/or Ca^+^ channels with outcomes depending on cell type (Duran-Gonzalez et al. 2013; Rice et al. 2014). Other channels that have been implicated with Aβ pathology could also contribute to the observed changes in firing. For instance, Kv4.3 channels, encoding A-type K^+^ currents (Molineux et al. 2005; Sun et al. 2011), the small-conductance, calcium-activated K^+^ channels (Greffrath et al. 2004; Yen et al. 1999), or the large-conductance, calcium-activated K^+^ channels (Brenner et al. 2005; Faber and Sah 2002; Ye et al. 2010), are among the candidates.

In addition to restoring Aβ-dependent changes in firing in Tg2576, RSG treatment in wild-type resulted in reduced firing and increased V_trh_ in type I granule cells and an increase in R_in_ in type II granule cells. These effects might be reconciled with direct action of RSG on ion channels, independent from PPARγ agonism (Ahn et al. 2007; Pancani et al. 2009; Wu et al. 2000).

#### Restoration of DG function through activation of the PPARγ axis: implications for memory enhancement in early AD.

The DG plays a fundamental role in memory formation through integration and consolidation of redundant, perforant path inputs and conversion into spatiotemporal patterns of sparse activity in the CA3 hippocampal area (Palmer and Good 2011). These intricate and substantive processes rely heavily on the functional integrity of DG granule neurons. Mature and adult-born, self-renewing granule neurons dynamically integrate into the DG circuit (Aimone et al. 2011; Nakashiba et al. 2012; Piatti et al. 2011; Sahay et al. 2012). As part of this process, synaptic inputs, intrinsic firing, and modes of activity-dependent plasticity are replenished continuously through birth of new neurons that progressively provide the requisite balance of immature and mature neurons for proper pattern separation that underlies the role of the DG in context discrimination (Jinno 2011; Piatti et al. 2011; van Praag et al. 2002). This makes the DG an exceptionally vulnerable brain region to Aβ-related pathologies and a hallmark of early AD (Palmer and Good 2011).

Links among impaired brain insulin signaling, cognition, and AD are well established in rodent models (Bosco et al. 2011; Rodriguez-Rivera et al. 2011; Schioth et al. 2012; Talbot et al. 2012) and humans (Craft et al. 2012; Risner et al. 2006; Sato et al. 2011; Watson et al. 2005). Evidence indicates a concerted activity of the PPARγ axis in ameliorating AD pathology by acting on the DG circuit (Denner et al. 2012; Nenov et al. 2014). Activation of the PPARγ axis improves hippocampal-related memory in some humans, with early AD and mouse models for AD-like amyloidosis (Escribano et al. 2010; Hoefer et al. 2008; Hort et al. 2007; Jahrling et al. 2014; Pedersen et al. 2006; Risner et al. 2006; Rodriguez-Rivera et al. 2011; Watson et al. 2005). In the Tg2576 mouse model for AD amyloidosis, PPARγ agonists restore presynaptic function and short-term synaptic plasticity at mPP to DG granule neuron synapses (Nenov et al. 2014) and rescue ERK signaling pathways that subserve hippocampus-dependent cognition (Ahi et al. 2004; Atkins et al. 1998; Denner et al. 2012; McGaugh 2000; Sweatt 2004).

Overall, our studies reinforce the complex role of the DG circuit in early AD pathology and suggest that in addition to restoring synaptic function (Denner et al. 2012; Nenov et al. 2014), RSG might improve cognition by ameliorating aberrant firing properties of DG cells and rectifying the output function of the DG circuit. These novel findings provide a deeper understanding of the basic mechanisms underlying early AD cognitive deficits and new opportunities for targeted therapeutic interventions.

## GRANTS

Support for this study was provided by the Institute for Translational Sciences at The University of Texas Medical Branch and, in part, by a Clinical and Translational Science Award (UL1TR000071; to F. Laezza) from the National Center for Advancing Translational Sciences; start-up funds from the Department of Pharmacology & Toxicology (to F. Laezza); and National Institute of Allergy and Infectious Diseases (AG031859-01A1; to K. T. Dineley and L. Denner). Additional funding was provided to K. T. Dineley and L. Denner by Bright Focus, The Sealy Foundation for Biomedical Research, and a gift from J & W Mohn. The Cullen Trust for Health Care provided funds to the Mitchell Center for Neurodegenerative Diseases.

## DISCLOSURES

No conflicts of interest, financial or otherwise, are declared by the author(s).

## AUTHOR CONTRIBUTIONS

Author contributions: M.N.N., L.D., K.T.D., and F.L. conception and design of research; M.N.N. performed experiments; M.N.N., F.T., and F.L. analyzed data; M.N.N., F.T., and F.L. interpreted results of experiments; M.N.N., F.T., and F.L. prepared figures; M.N.N. and F.L. drafted manuscript; M.N.N., F.T., L.D., K.T.D., and F.L. edited and revised manuscript; M.N.N., F.T., L.D., K.T.D., and F.L. approved final version of manuscript.
